# Editorial: Prognostic and predictive factors in autoimmune connective tissue disorders

**DOI:** 10.3389/fimmu.2024.1465572

**Published:** 2024-07-29

**Authors:** Ilaria Mormile, Mohammed Osman, Francesca Wanda Rossi

**Affiliations:** ^1^ Department of Translational Medical Sciences, University of Naples Federico II, Naples, Italy; ^2^ Division of Rheumatology, Department of Medicine, University of Alberta, Edmonton, AB, Canada; ^3^ Center for Basic and Clinical Immunology Research (CISI), University of Naples Federico II, World Allergy Organization (WAO) Center of Excellence, Naples, Italy

**Keywords:** biomarkers, autoimmunity, autoimmune diseases, adaptive immunity, innate immunity, inflammation, connective tissue disorders

Autoimmune connective tissue diseases (CTD) are a complex group of diseases involving multiple organs and resulting in devastating visceral complications and even death. One of the most recognized challenges related to them is their variable disease courses that range from indolent ones to rapidly progressive clinical disease trajectories. This disease-associated heterogeneity is associated with differences in the severities of organ involvement, disease-specific damage accrual, autoantibody profiles, response(s) to treatment, and, ultimately, long-term survival. Hence, prognostic biomarkers that are ideally associated with the pathogenesis of these diseases are desperately needed.

In this Research Topic, “Prognostic and Predictive Factors in Autoimmune Connective Tissue Disorders”, submissions were solicited for original research articles, brief research reports, and review papers with a particular emphasis on novel serological biomarkers of disease severity, evaluation of gene expression and protein levels of inflammatory mediators, adhesion proteins, cytokines, and pro-resolving molecules, recent applications of routinary laboratory parameters and instrumental techniques for assessing disease severity and systemic involvement, and development of new multi-parametrical disease activity score for autoimmune CTD. Since the Research Topic was open to submission, 39 manuscripts were submitted, of which 27 were rejected, and 12 were published. The Research Topic was viewed about 20,500 times, highlighting the scientific community’s interest in the subject, and its relevance to patients.

An original research article by Atzeni et al. evaluated the soluble receptor for advanced glycation end products (RAGE) and its ligand high mobility group box 1 (HMGB1) as a potential predictor of pulmonary arterial hypertension (PAH) in systemic sclerosis (SSc). SSc (in general) and PAH (in particular) are characterized by a systemic progressive obliterative vasculopathy. Both interstitial lung disease (ILD) and PAH significantly impact the long-term survival of SSc patients. Early in the disease, SSc patients may develop pulmonary hypertension which may stem from ILD or PAH. Notably, there are no biomarkers can preferentially identify patients developing these complications. The authors demonstrated that high systemic levels of sRAGE in SSc patients at baseline might be useful for predicting new-onset PAH. Indeed, sRAGE were much lower in patients who developed ILD, suggesting that elevated sRAGE may be more specific for PAH in SSc patients. Also, sRAGE levels were inversely proportional to patient survival. Future studies assessing a role for sRAGE in promoting vasculopathy (e.g., cardiovascular disease), or in resistant pulmonary arteries may provide added insights related to its role in the pathogenesis of SSc as both complications are more common in this group of patients (compared to age and sex-matched controls).

Zheng et al. focused on developing and validating a new diagnostic prediction model using machine learning and differential expression of ENHO and NOX4 for the progression of skin fibrosis in skin from patients with SSc. Increased ENHO and NOX4 are associated with immune cell activation and fibroblast, respectively, both of which play a pivotal role in the pathogenesis and progression of SSc. The goal set by the authors is valuable since patients with rapidly progressive forms of diffuse SSc have a small window for clinical intervention with immunomodulation. Hence, early identification of these patients may result in improved survival, whereas poor identification of these patients may result in increased disease-associated morbidity and poor outcomes.

Other notable contributions related to the development of predictive biomarkers were provided by Zhang et al. and Wang et al. Using peptidomics, Zhang et al. were able to identify three peptides (sequence: DSGEGDFLAEGGGVRGPR, NGFKSHAL, ISEQFTAMFR) in sera from patients with AS that promoted the proliferation of fibroblasts derived from patients with AS. This is highly relevant to the pathogenesis of AS, as fibroblasts in AS are known to promote one of the earliest changes related to new bone formation. Further to this, Wang et al. performed a two-sample Mendelian randomization and meta-analysis and found that elevated levels of serum lipid metabolites may contribute to the development of autoimmune diseases.

Other authors in this Research Topic focused on patients with severe outcomes related to dermatomyositis (DM). Rapidly progressive ILD (RP-ILD) is a major cause of death in patients with anti-melanoma differentiation-associated gene 5 positive DM (anti-MDA5+DM). Wang et al. used Cox proportional hazards models to identify risk factors of RP-ILD. In particular, the authors selected four independent risk factors and then created a new RP-ILD risk prediction model, which was designated as the CROSS score (comprised of elevated C-reactive protein (CRP) levels, anti-Ro52 antibody positivity, male sex, and short disease duration < 3 months from disease onset). Predictors of RP-ILD in anti-MDA5+ DM were also evaluated by Li et al., along with assessing prognostic factors. The analysis conducted in 73 MDA5+ DM patients showed that elevated lactate dehydrogenase (LDH) and elevated prognostic nutritional index (PNI) were independent prognostic factors. Moreover, an elevated LDH was an independent risk factor for RP-ILD (> 356 U/ml with a mortality rate of approximately 65% after 3 years). In another research article by Dong et al., the anti-carbamylation protein antibodies (anti-CarPA) assessment was proposed as a prediction biomarker for the development of ILD in patients with PM/DM, rheumatoid arthritis, and primary Sjögren’s syndrome. Finally, Ji et al. compared clinical characteristics and risk factors for mortality between clinically amyopathic dermatomyositis (CADM) and classic DM (CDM) to clarify the distribution and impact of anti-MDA5 antibodies in patients with these conditions. The retrospective case-control study, which included 330 patients showed that CADM patients display higher anti-MDA5 antibody levels, worse symptoms, and worse prognosis, often requiring an earlier and more aggressive treatment than CDM.

In an intriguing study by Wang et al., the authors determined the probability of response to treatment in lupus nephritis by closely examining the tubulointerstitial macrophage infiltrations in 430 patients at the time of the renal biopsy. Importantly, the density of tubulointerstitial macrophage infiltration is a favorable independent predictor for treatment response (with an AUC of 0.78). Hence, this simple predictive biomarker could be added to the clinicopathological data at the time of biopsy for improved risk stratification. Renal involvement influences the prognosis of several other autoimmune conditions. Further to this, Uchida et al. found that the renal risk score (RRS), which is comprised of the percentage of normal glomeruli, the frequency of tubular atrophy/interstitial fibrosis, and the estimated glomerular filtration rate, is a reliable predictor of renal survival in Japanese patients with ANCA-associated glomerulonephritis.

This Research Topic also included novel observations relevant to patients with autoimmune CTDs. For instance, Maranini et al. presented the first case of a patient with eosinophilic granulomatosis with polyangiitis (EGPA) and middle lobe syndrome, which completely resolved through fiberoptic bronchoscopy, underlying the concept that the identification of this condition in EGPA patients may be essential to guide their management, treatment, and prognostication. Further to this, Xian et al. investigated the correlation between systemic erythematous lupus and Graves’ disease. In their study, they suggested that the presence of SLE may potentially increase the risk of Grave’s disease, highlighting a unifying common pathway related to immune dysregulation.

In conclusion, the articles published in this Research Topic have led to novel insights related to the prediction of autoimmune CTDs and their sequelae. They also linked previously unrelated clinical observations with laboratory ones to provide additional information related to the diagnosis and prognosis of patients with these diseases. Together, these developments may lead to personalized treatment strategies which may improve the care of patients with these devastating autoimmune diseases.

## Author contributions

IM: Writing – original draft, Validation. MO: Writing – review & editing, Validation. FR: Writing – review & editing, Validation.

